# The diagnosis of colorectal cancer in patients with symptoms: finding a needle in a haystack

**DOI:** 10.1186/1741-7015-7-18

**Published:** 2009-04-17

**Authors:** Robert H Fletcher

**Affiliations:** 1 Harvard Medical School, Boston, Massachusetts, USA

## Abstract

Patients often see primary care physicians with symptoms that might signal colorectal cancer but are also common in adults without cancer. Physicians and patients must then make a difficult decision about whether and how aggressively to evaluate the symptom. Favoring referral is that missed diagnoses lead to unnecessary testing, prolonged uncertainty, and continuing symptoms; also, the physician will suffer chagrin. It is not clear that diagnostic delay leads to progression to a more advanced stage. Against referral is that proper evaluation includes colonoscopy, with attendant inconvenience, discomfort, cost, and risk. The article by Hamilton et al, published this month in BMC Medicine, provides strong estimates of the predictive value of the various symptoms and signs of colorectal cancer and show how much higher predictive values are with increasing age and male sex. Unfortunately, their results also make clear that most colorectal cancers present with symptoms with low predictive values, < 1.2%. Models that include a set of predictive variables, that is, risk factors, age, sex, screening history, and symptoms, have been developed to guide primary prevention and clinical decision-making and are more powerful than individual symptoms and signs alone. Although screening for colorectal cancer is increasing in many countries, cancers will still be found outside screening programs so primary care physicians will remain at the front line in the difficult task of distinguishing everyday symptoms from life-threatening cancer.

## Commentary

Patients often see primary care physicians for symptoms that might signal colorectal cancer, raising difficult questions. Which patients should be evaluated? How aggressively should they be worked up? If another cause for the symptom is found, such as hemorrhoids for rectal bleeding, should that set the matter to rest or should a cancer diagnosis still be pursued?

Table 1 lists 15 symptoms of colorectal cancer that have been suggested in textbooks and supported by research evidence [1]. Unfortunately, many of these same symptoms, especially constipation and fatigue, are common in patients who do not have colorectal cancer. True, some clinical presentations, such as bowel obstruction or severe abdominal or rectal pain, are sufficiently unusual and dramatic events that they would prompt quick evaluation in any case. However, most symptoms of colorectal cancer are not so compelling. As a result, first-contact physicians are in the familiar position of looking for a needle in a haystack.

**Table 1:** Presenting symptoms and signs for 194 patients with colorectal cancer

**Symptom**	**Percentage of patients**
Fecal occult blood test positive	77

Rectal bleeding	58

Anemia*	57

Abdominal pain	52

Weight loss	39

Anorexia	27

Constipation	27

Altered stools	25

Fatigue	25

Diarrhea	22

Nausea and vomiting	22

Tenesmus	8

Mucus in stools	6

Rectal pain	5

Obstruction	4

Adapted from Majumdar et al. [1].

*Anemia = a hemoglobin of < 13.4 g/dl in men or < 12.3 g/dl in women.

The stakes are high on either side of the decision. If the diagnosis is missed the patient will undergo unnecessary testing, prolonged uncertainty, and continuing symptoms until the diagnosis is finally made. The clinician will suffer chagrin [2] and in some settings he or she might also worry about malpractice claims. On the other hand, proper evaluation, which involves complete visualization of the large bowel by colonoscopy, is a big undertaking, with the inconvenience of a day off work, the discomfort of bowel cleansing if not the procedure itself, financial costs to the patients or society, and a small risk of perforation, bleeding, or other complications [3].

Does diagnostic delay allow colorectal cancer to progress from a local to advanced stage, diminishing the possibility of cure? One might think so but the evidence is mixed and for the most part against this possibility [1,4-6]. The relationship between diagnostic delay and cancer stage or survival is at the very least complex. For example, a study of 777 consecutive colorectal cancer patients found that shorter duration of symptoms was associated with advanced tumor stage [4]. This makes sense because estimates of the transition time from localized to advanced colorectal cancers is measured in years, much longer than the time from symptoms to diagnosis in most patients. Also, advanced cancers causing bowel obstruction would be evaluated promptly while some slow-growing, localized tumors may not declare themselves (perhaps with systemic symptoms such as fatigue or weight loss) for many months. Therefore, while the other consequences of diagnostic delay are certainly in play, progression to more advanced stage may not be.

The article by Hamilton et al, published this month in BMC Medicine, advances the evidence base for early diagnosis of colorectal cancer [7]. The investigators were fortunate to have access to a large database of patients in general practices in the UK, with data on 23 candidate symptoms and signs of colorectal cancer (or surrogates for some of them, such as drugs prescribed for constipation). The authors calculated likelihood ratios for symptoms and signs from data on 5477 cases of colorectal cancer and controls matched for age, sex, and site. They then estimated the positive predictive values of these symptoms and signs by applying their likelihood ratios to national data on the incidence of colorectal cancer, using Bayes' theorem: prior odds × likelihood ratio = posterior odds (where the national incidence data was used to derive prior odds). This approach is sound if one is willing to assume that patients in the general practices and in the nation as a whole are comparable. Given the size of the database, the authors could describe the predictive value of each symptom and sign with clinically useful precision, even though colorectal cancer is uncommon. The authors analyzed the data and described their results with an admirable grasp of both the scientific issues and the clinical realities of colorectal cancer presentation in general practice.

The Hamilton study identified predictive variables that have long been part of clinical lore and more recently confirmed by clinical research. With their large sample size, the authors were able to go further to show how powerfully age, and to a lesser extent sex, affect predictive values for this condition. However, the authors admit that their study could not solve the problem of early diagnosis, mainly because most patients presented with symptoms with low predictive values. The presenting symptoms for 73% of colorectal cancer patients had predictive values of < 1.2%. Although two symptoms (rectal bleeding and change in bowel habits) had relatively high positive predictive values, they were uncommon. Therefore, physicians must still wrestle with referral decisions for the larger proportion of patients with symptoms only weakly associated with colorectal cancer.

As the authors suggest, a set of variables may predict better than individual symptoms and signs taken one at a time. Predictive models for colorectal cancer have been developed [8-12]. Some of these models are designed to guide primary prevention and include age and behavioral risk factors such as smoking, diet, obesity, and exercise. Others are for clinical cancer detection and include symptoms and signs, as well as age. Models posted on the US National Institutes of Health and Harvard Medical School's public websites [11,12] take screening history into account, as they should in settings such as the US where colorectal cancer screening has been widely practiced for years. Unfortunately, no model so far, whether for preventive care or clinical diagnosis, includes all of these predictors. Even if one did, it might not produce strong enough odds ratios to be as helpful in individual patients as in groups of patients [13,14].

It is always difficult to choose a reasonable threshold for further testing. Hamilton et al. assert that most would agree that positive predictive values in the 2.4% to 4.5% range, seen with rectal bleeding in men over 60 years, are high enough to warrant investigation whereas positive predictive values below 1.5% (as seen with constipation, diarrhoea, abdominal pain, and loss of weight) reflect 'low-risk symptoms'. Other general practitioners in the UK, or referral physicians, may have different opinions. Care with high costs and low yield may be considered worthwhile in one country but out of reach or profligate in others. Also, the implementation of predictive models are compromised to the extent that physicians feel uncomfortable missing any cancers, no matter how low the probability, and so make decisions using 'clinical judgment' even when powerful predictive models exist [15,16].

Will screening programs make the clinical diagnosis of colorectal cancer a challenge of the past? Screening for colorectal cancer has been shown to be effective and is being implemented in many countries [17]. In the US, nearly two-thirds of adults are being screened [18] and colonoscopy is becoming the test of choice [19]. Other countries have for the most part chosen fecal occult blood testing for average risk adults [17]. Is it not likely that in the future screening will find most cancers, or lead to their prevention by removing polyps, so that the need for clinical diagnosis will all but disappear. Probably not because of the net effects of insensitive tests, refusal to be screened, and new cancers in the interval between screenings. Therefore, primary care physicians will remain at the front line in the difficult task of distinguishing everyday symptoms from life-threatening cancer.

## Pre-publication history

The pre-publication history for this paper can be accessed here:


